# Analysing *GCN4 *translational control in yeast by stochastic chemical kinetics modelling and simulation

**DOI:** 10.1186/1752-0509-5-131

**Published:** 2011-08-18

**Authors:** Tao You, Ian Stansfield, M Carmen Romano, Alistair JP Brown, George M Coghill

**Affiliations:** 1 School of Natural and Computing Sciences, University of Aberdeen, Institute of Complex System and Mathematical Biology, Aberdeen, UK; 2 School of Medical Sciences, University of Aberdeen, Institute of Medical Sciences, Aberdeen, UK; 3 School of Natural and Computing Sciences, University of Aberdeen, Computing Science Department, Aberdeen, UK; 4 AstraZeneca, Mereside, Alderley Park, Macclesfield, UK

**Keywords:** mRNA translation, *GCN4*, Gillespie algorithm, stochastic model

## Abstract

**Background:**

The yeast *Saccharomyces cerevisiae *responds to amino acid starvation by inducing the transcription factor Gcn4. This is mainly mediated *via *a translational control mechanism dependent upon the translation initiation eIF2·GTP·Met-tRNA_i_^Met ^ternary complex, and the four short upstream open reading frames (uORFs) in its 5' mRNA leader. These uORFs act to attenuate *GCN4 *mRNA translation under normal conditions. During amino acid starvation, levels of ternary complex are reduced. This overcomes the *GCN4 *translation attenuation effect via a scanning/reinitiation control mechanism dependent upon uORF spacing.

**Results:**

Using published experimental data, we have developed and validated a probabilistic formulation of *GCN4 *translation using the Chemical Master Equation (Model 1). Model 1 explains *GCN4 *translation's nonlinear dependency upon uORF placements, and predicts that an as yet unidentified factor, which was proposed to regulate *GCN4 *translation under some conditions, only has pronounced effects upon *GCN4 *translation when intercistronic distances are unnaturally short. A simpler Model 2 that does not include this unidentified factor could well represent the regulation of a natural *GCN4 *mRNA. Using parameter values optimised for this algebraic Model 2, we performed stochastic simulations by Gillespie algorithm to investigate the distribution of ribosomes in different sections of *GCN4 *mRNA under distinct conditions. Our simulations demonstrated that ribosomal loading in the 5'-untranslated region is mainly determined by the ratio between the rates of 5'-initiation and ribosome scanning, but was not significantly affected by rate of ternary complex binding. Importantly, the translation rate for codons starved of cognate tRNAs is predicted to be the most significant contributor to the changes in ribosomal loading in the coding region under repressing and derepressing conditions.

**Conclusions:**

Our integrated probabilistic Models 1 and 2 explained *GCN4 *translation and helped to elucidate the role of a yet unidentified factor. The ensuing stochastic simulations evaluated different factors that may impact on the translation of *GCN4 *mRNA, and integrated translation status with ribosomal density.

## Background

Reprogramming gene expression is an important means for cells to adapt to environmental changes. In eukaryotes, gene expression is regulated at multiple levels, including transcription, RNA splicing and translation. Translational control mechanisms, particularly acting at the level of translation initiation, can be a primary point of regulation for certain genes. The yeast *GCN4 *gene is one such example. It encodes a transcription factor that regulates expression of genes encoding amino acid biosynthetic (and other) enzymes. As such, it plays a central role in the amino acid starvation or GCN response [1,2].

**Figure 1:**
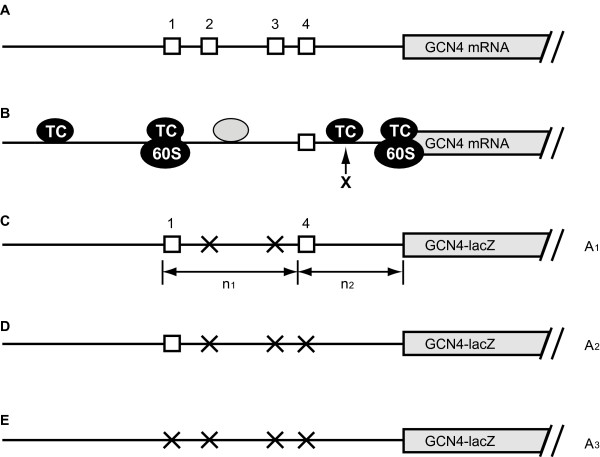
***GCN4 *translational control**. The positions of the uORFs (open boxes) in the 5' leader sequence are drawn roughly to scale. The main *GCN4 *ORF is depicted partially. Point mutations that remove the start codon of a uORFs are labelled by cross. (A) Wild type *GCN4 *mRNA structure. (B) Cartoon of *GCN4 *mRNA translation. (C-E) *GCN4-lacZ *constructs that were used to investigate *GCN4 *translation under repressing and derepressing conditions, the activities of which are denoted as *A*1, *A*2 and *A*3, respectively.

*GCN4 *mRNA translation is regulated through an unusually long 5'-leader region (591 nucleotides in length), which contains four short upstream open reading frames (uORFs) (Figure 1A) [2]. While uORFs in 5' leaders can frequently attenuate translation of the downstream open reading frame, some allow ribosomes to resume scanning following uORF translation. This is dependent on the nature of a roughly 15-nucleotide long sequence immediately downstream of the uORF stop codon [2]. *GCN4 *uORF1 and uORF2 have this property, and are relatively weak barriers that allow nearly half of the ribosomes to remain on the *GCN4 *mRNA after their translation, while uORF3 and uORF4 are more inhibitory, causing nearly all of the ribosomes to disassociate from the *GCN4 *mRNA after their translation [2]. A recent study further suggests that after uORF1 translation, the ribosome dissociation from the mRNA is prevented by a mechanism involving eIF3 interaction with the mRNA [3].

At the beginning of *GCN4 *mRNA translation, a 43S ribosomal subunit, incorporating an eIF2·GTP·Met-tRNA_i_^Met ^ternary complex (TC), scans from the 5' end of the mRNA to initiate translation at uORF1. Following uORF1 translation termination, about half of the 40S subunits remain on the mRNA and resume scanning. When amino acids are abundant, the concentration of ternary complex is relatively high, these scanning 40S ribosomal subunits efficiently re-acquire ternary complex after uORF1 translation, forming active 48S preinitiation complexes. These 48S complexes reinitiate (i.e. recognise and subsequently translate) at downstream uORFs 3 and 4, which have 3' sequence contexts that promote ribosome release. This restricts the supply of ribosomes to the main *GCN4 *ORF and attenuates its translation. Hence, Gcn4 protein production is low under amino acid replete conditions (Figure 1B).

When yeast cells are starved of amino acids, phosphorylation of eIF2 by the Gcn2 kinase causes a reduced abundance of eIF2· GTP [2], and a consequential reduction in the concentration of ternary complex. 40S subunits scanning downstream of uORF1 have a reduced chance of re-acquiring ternary complex. Instead, 40S subunits frequently re-associate with eIF2·GTP·Met-tRNA_i_^Met^, only when scanning has progressed past uORFs 3 and 4, but before the main *GCN4 *AUG codon. The translation of the main *GCN4 *ORF under starvation conditions elevates Gcn4 synthesis by about 34-fold, which leads to the activation of amino acid biosynthetic genes [4].

**Figure 2:**
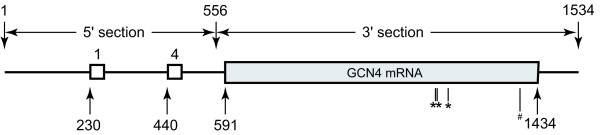
**The structure of *GCN4 *mRNA considered in the stochastic model**. Numbers indicate the positions of start codon of the uORFs, start and end of *GCN4*, and the end of the mRNA. Histidine codons in *GCN4 *are highlighted (* CAU, # CAC). The mRNA is cut into two sections, namely the 5'-(1~555 nt) and the 3'-(556~1534 nt) fractions.

It was reported that *GCN4 *mutants lacking uORF2 and uORF3 displayed essentially normal *GCN4 *translational behaviour [2]. This is because those ribosomes blocked by uORF2 and uORF3 would be blocked instead by uORF4, if those two uORFs were removed. For the sake of simplicity, only uORF1 and uORF4 are considered in the rest of our discussions in this paper. The structure of such a *GCN4 *mRNA is depicted in Figure 2.

uORF spacing, and its effect on ternary complex re-acquisition, is thus central to the *GCN4 *translational control mechanism. Intuitively, given a constant scanning speed, the time it takes for a 40S subunit to reach uORF4 from uORF1 should scale linearly with the corresponding intercistronic distance between uORF1 and uORF4. Naively, one would assume that the proportion of ribosomes that translate uORF4 is linearly dependent upon this distance, since the 40S subunits have more time to bind the ternary complex. However, it was found that this proportion depends nonlinearly upon the intercistronic distance [4]. This unexplained observation motivated our use in this study of mathematical modelling of this stochastic process as an important tool to analyse the *GCN4 *control. Naturally, we chose to use a stochastic theoretical framework to address these issues.

Previous work from the Hinnebusch laboratory on how unnaturally short intercistronic distances between the upstream and main ORFs affect the rate of reinitiation at the main *GCN4 *ORF has implicated an additional unidentified factor (Factor X) in the recognition of the *GCN4 *start site (Figure 1B) [4]. This factor is not required for uORF4 start codon selection. In contrast to ternary complex, its levels are low when amino acids are replete, and its levels are high under amino acid starvation conditions. This factor can help explain the difference in translational behaviours of uORF4 and the main *GCN4 *ORF under repressing and derepressing conditions. Factor X could be an unknown protein, or an identified initiation factor that is involved in start codon selection (see Discussion for more details). In this study, probabilistic modelling was used to evaluate how Factor X affects translation of *GCN4 *mRNA with different intercistronic distances.

Our aim in this paper has been to develop a quantitative understanding of *GCN4 *mRNA translational control, taking into account stochastic effects, and to use that model to understand some hitherto unexplained experimental observations. Previously we reported a simple probabilistic model of *GCN4 *mRNA translation [5]. Here, we constructed a comprehensive probabilistic model that encompasses more mechanistic details based on Chemical Master Equation (Model 1). This approach gives the model a rigorous theoretical basis. This model was simplified to form a probabilistic Model 2. We used Model 2 to estimate ternary complex levels under repressing (replete) and derepressing (starvation) conditions. Based on these values, we developed a stochastic model (Model 3) to include the effects of steric hindrance caused by scanning ribosomes. Using the Gillespie algorithm, we performed stochastic simulations to investigate how translation of *GCN4 *mRNA is affected by different parameters, including 5'-loading of ribosomes and the scanning rate.

## Methods

### Translation of uORF4 and *GCN4 *protein coding ORF

Prior to model construction, we first briefly review the different translational behaviours of uORF4 and *GCN4*. These findings are essential to develop Model 1. For the sake of discussion, we refer in the rest of the paper to the intercistronic distance between uORF1 and uORF4 as *n*_1_, and the distance between uORF4 and the main *GCN4 *ORF as *n*_2_. To investigate uORF4 reinitiation, Grant *et al*. [4] made pairs of *GCN4-lacZ *constructs, each with different (*n*_1_+*n*_2_) distances (Figure 1C, D). One member of each pair has a mutated uORF4 start codon. The *GCN4*-*lacZ *activity for each construct plus and minus uORF4 is denoted by *A*_1 _and *A*_2_, respectively. The probably that uORF4 is translated by a 40S subunit scanning downstream of uORF1 can be estimated for both repressing and derepressing conditions:

(1) $$P_{uORF4}^{obs}\left(n_{1},n_{2}\right)=\frac{A_{2}-A_{1}}{A_{2}}\times 100\%$$

$P_{uORF4}^{obs}\left(n_{1},n_{2}\right)$ is a relative percentage, and it is sensitive to ternary complex levels.

**Figure 3:**
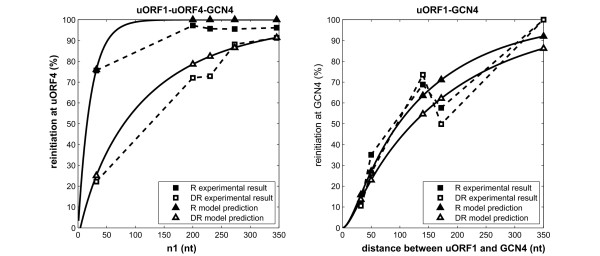
**Intercistronic distances affect the percentage of ribosomes that reinitiate at uORF4 and *GCN4 *(Model 1)**. Published experimental data (from [5]: repressing: black square; derepressing: white square. A: $P_{uORF4}^{obs}\left(n_{1},n_{2}\right)$ B: $P_{GCN4}^{obs}\left(n_{1},n_{2}\right)$.) and model predictions (repressing: black triangle; derepressing: empty triangle. A: $P_{uORF4}^{theo}\left(n_{1},n_{2}\right),$ B: $P_{GCN4}^{theo}\left(n_{1},\;n_{2}\right)$.) were plotted together.

Under repressing conditions (amino acid replete), nearly 80% of ribosomes translate uORF4 and abandon the mRNA after scanning just 32 nucleotides, whereas only about 20% of the ribosomes do so under derepressing conditions (amino acid starvation) (Figure 3A).

To study translation of *GCN4 *main ORF, Grant *et al*. [4] further made different pairs of *GCN4-lacZ *constructs as shown in Figure 1D and 1E. Again, a pair of constructs was made for each (*n*_1_+*n*_2_) distance, whereby one construct had a functional uORF1 and its pair had a non-functional uORF1 due to a point mutation in start codon. They measured the *GCN4*-*lacZ *activity of each pair of constructs (*A*_2 _and *A*_3 _in Figure 1, respectively), and estimated the percentage of *GCN4 *ORF reinitiation by

(2) $$P_{GCN4}^{obs}(n_{1},n_{2})=\left(\frac{A_{2}}{A_{3}}/\frac{A_{2}^{0}}{A_{3}^{0}}\right)\times 100\%,$$

where $A_{2}^{0}$ and $A_{3}^{0}$ are the activities of the constructs with the wild type uORF1-*GCN4 *distance.

Grant *et al*. [4] showed that $P_{GCN4}^{obs}\left(n_{1},n_{2}\right)$. and $P_{uORF4}^{obs}\left(n_{1},n_{2}\right)$ depend on (*n*_1_+*n*_2_) in markedly different fashions (Figure 3). $P_{uORF4}^{obs}\left(n_{1},n_{2}\right)$ is sensitive to the amino acid availabilities (Figure 3A). In contrast, $P_{GCN4}^{obs}\left(n_{1},n_{2}\right)$ exhibits similar (*n*_1_+*n*_2_) dependency under both repressing and derepressing conditions, resulting in the two curves almost coinciding with each other. After ruling out the possibility that mRNA secondary structures lower scanning rates in the uORF1-*GCN4 *constructs, the existence of a Factor X was hypothesized to explain this phenomenon [4]. To reinitiate at the main *GCN4 *ORF, a 40S subunit needs to bind an extra Factor X in addition to ternary complex during its scanning. This factor is not required for uORF4 reinitiation. Factor X has low activities under amino acid replete conditions, and assumes high activities in response to amino acid starvation. In what follows, we use stochastic modelling to examine the contributions of both ternary complex and the hypothetical Factor X on *GCN4 *control.

### Model 1 hypotheses

We applied stochastic chemical kinetics to model *GCN4 *mRNA translation. First we developed a probabilistic model of the likelihood that a ribosome either translates the inhibitory uORF4 or the main *GCN4 *ORF under repressing and derepressing conditions. Several simplifications were made to construct Model 1.

Recent studies suggest that reverse scanning is negligible [6,7]. It is therefore not considered in the model. Ribosome scanning is a highly efficient process [6,8]. Hence, we assume that ribosomes do not abort scanning. We further assume that ribosomes scan at a constant speed. In principle, the ternary complex can dissociate from the 40S ribosomal subunit. However, it was recently reported that this is a slow process *in vitro *[9]. Therefore, this rare event is not considered. Finally, we assume that there is no "leaky scanning" (i.e. a 48S complex recognises and translates the immediately next ORF it encounters), and that all ribosomes dissociate from the mRNA after translating uORF4. It is worth noting that all reactions, including scanning, are expressed using a common dimension nt/s in this work. For more detailed discussions, please see Supporting Information section S0 (Additional file 1).

### Derivation of a simple model from Chemical Master Equation

First we developed a probabilistic model of the likelihood that a ribosome either translates the inhibitory uORF4 or the main GCN4 ORF under repressing and derepressing conditions.

The regulation of the *GCN4 *is crucially dependent upon the distances *n_1 _*and *n_2_*. Previous detailed analysis of the dependencies of *GCN4 *ORF translation on *n_1 _*and *n_2 _*revealed evidence that a post-uORF1 scanning ribosome has to acquire not only ternary complex, but also an unidentified Factor X to become competent to initiate at the *GCN4 *AUG [4]. In what follows, we use stochastic modelling to examine the contribution of the hypothetical Factor X on *GCN4 *control, and the interplay between Factor X and ternary complex.

Here, we first consider a simple scenario. The conclusion drawn from the analysis of this simple example is important to construction of Model 1. Suppose a 40S ribosomal subunit scans downstream of uORF1, and we are concerned with the probability of this 40S subunit reaching a certain distance without binding any factor. This system includes two reactions: a unidirectional scanning reaction in which a 40S subunit moves forward by 1 nucleotide, and binding of an additional factor. This factor could be ternary complex, or Factor X. Because the system only concerns 40S subunit, binding an additional factor changes its identity and leads to the disappearance of 40S subunit.

(3) $$40S\left(n\right)\overset{a_{S}}{\underset{}{\to }}40S\left(n+1\right)$$

(4) $$40S\left(n\right)\overset{a_{D}}{\underset{}{\to }}$$

where *a_S _*and *a_D _*are propensity functions, which are equivalent to reaction rates in the conventional deterministic kinetics.

Consider the probability of a ribosome at a particular position *n *nucleotide downstream of uORF1 at time *t*, *P*(*n*, *t*). Consider an infinitesimal time interval *δt *that is so short that only one of the following two events is allowed to happen: either a ribosome moves into this site if this site is originally empty, or this site is originally occupied by a ribosome which does not move during *δt*. Hence, the probability of a ribosome at position *n *at a later time *t *+ *δt*, *P*(*n*, *t *+ *δt*), is equal to the sum of the probabilities of these two independent events:

$$\begin{array}{c} P\left(n,t+\delta t\right)=\\ P\left(n-1,t\right)a_{S}\delta t+P\left(n,t\right)\left(1-a_{s}\delta t\right)\left(1-a_{D}\delta t\right) \end{array}$$

Here we use the fist order approximation of time. Hence, the term involving can be ignored. Rearranging this equation, we can write down the Chemical Master Equation,

(5) $$\frac{\partial P\left(n,t\right)}{\partial t}=a_{s}\left[P\left(n-1,t\right)-P\left(n,t\right)\right]-a_{D}P\left(n,t\right).$$

Introducing a generating function $G\left(s,t\right)=\overset{\infty }{\underset{n=0}{\sum }}s^{n}P\left(n,t\right)$, equation 5 becomes

$$\begin{array}{c} \frac{\partial G\left(s,t\right)}{\partial t}=\overset{\infty }{\underset{n=0}{\sum }}s^{n}\frac{\partial P\left(n,t\right)}{\partial t}\\ =\overset{\infty }{\underset{n=0}{\sum }}s^{n}\left\{a_{S}\left[P\left(n-1,t\right)-P\left(n,t\right)\right]-a_{D}P\left(n,t\right)\right\}.\\ =a_{S}\left(s-1\right)G\left(s,t\right)-a_{D}G\left(s,t\right)\\ =G\left(s,t\right)\left[a_{S}\left(s-1\right)-a_{D}\right] \end{array}$$

This equation is soluble by integrating over time *t*,

$$G\left(s,t\right)=\exp\left\{\left[a_{S}\left(s-1\right)-a_{D}\right]t\right\}\cdot G\left(s,0\right).$$

At time 0, the probability of a ribosome being at the first nucleotide downstream of uORF1 (*i.e*. nucleotide 0) is 1, and the probability of a ribosome at any other position is 0. According to its definition, *G*(*s*, *0*) = *1*, and the previous equation becomes

$$G\left(s,t\right)=\exp\left\{\left[a_{S}\left(s-1\right)-a_{D}\right]t\right\}.$$

Consider the definition of *G*(*s*, *t*) and rewrite exp(a_S_st) by Taylor expansion,

$$\begin{array}{c} \overset{\infty }{\underset{n=0}{\sum }}s^{n}P\left(n,t\right)=\\ \exp\left[-\left(a_{S}+a_{D}\right)t\right]\cdot \overset{\infty }{\underset{n=0}{\sum }}\frac{a_{s}^{n}\cdot s^{n}\cdot t^{n}}{n!} \end{array}$$

Therefore,

(6) $$P\left(n,t\right)=\frac{a_{S}^{n}\cdot t^{n}}{n!}\exp\left[-\left(a_{S}+a_{D}\right)t\right].$$

This simple system exhibits a Poisson-like probability distribution function. The probability of having a ribosome at a particular position in the mRNA, also known as the positional marginal probability, can be consequently obtained by integrating the joint probability density function [6] over time,

(7) $$P\left(n\right)=\int_{t=0}^{\infty }P\left(n,t\right)dt={\left(\frac{a_{S}}{a_{S}+a_{D}}\right)}^{n}.$$

Alternatively, equation 7 can be directly obtained from Gillespie algorithm [10]. In a system in which a ribosome moves at rate *a_S _*and disappears at rate *a_D_*, the probability that the scanning reaction happens is equal to propensity function of scanning (*a_S_*) divided by the summation of propensity function of all possible reactions (*a_S _*+ *a_D_*). Consequently, the probability of a ribosome scanning *n *nucleotides without binding a ternary complex is the probability of consecutively selecting *n *times the scanning reaction, the same as defined in equation 7. This is an important intermediate conclusion that will be used in model developments that follow. Next, we derive a mechanistic Model 1 that considers both ternary complex and Factor X.

### Probabilistic Model 1 formulation

The process of *GCN4 *ORF reinitiation for the construct depicted in Figure 1D is divisible into three sub-processes. First, the 40S ribosomal subunit scans along the mRNA devoid of ternary complex and Factor X. This is followed by binding of the first factor, which could be either ternary complex or Factor X [4]. Then the ribosome acquires the second factor before traversing the remaining distance. The probability of assembling both factors is the product of the probabilities of the three individual sub-processes. It is unknown whether bindings of the two factors are cooperative. As a simplification, it is assumed that these reactions are not cooperative, e.g. binding of one factor does not change the rate of binding the other factor. This would reduce the number of unknown parameters to a tractable problem. The system consists of the following reactions.

(8) $$40S\left(n\right)\overset{a_{TC}}{\underset{}{\to }}40S\left(n\right)\cdot TC$$

(9) $$40S\left(n\right)\cdot X\overset{a_{TC}}{\underset{}{\to }}40S\left(n\right)\cdot TC\cdot X$$

(10) $$40S\left(n\right)\overset{a_{X}}{\underset{}{\to }}40S\left(n\right)\cdot X$$

(11) $$40S\left(n\right)\cdot TC\overset{a_{X}}{\underset{}{\to }}40S\left(n\right)\cdot TC\cdot X$$

(12) $$40S\left(n\right)\overset{a_{S}}{\underset{}{\to }}40S\left(n+1\right)$$

(13) $$40S\left(n\right)\cdot TC\overset{a_{S}}{\underset{}{\to }}40S\left(n+1\right)\cdot TC$$

(14) $$40S\left(n\right)\cdot X\overset{a_{S}}{\underset{}{\to }}40S\left(n+1\right)\cdot X$$

(15) $$40S\left(n\right)\cdot TC\cdot X\overset{a_{S}}{\underset{}{\to }}40S\left(n+1\right)\cdot TC\cdot X$$

According to equation 7, the probability of scanning *i *times before the ribosome binds any factor is *P_S1 _*= [*a_S_*/(*a_S _*+ *a_TC _*+ *a_X_*)]*^i^*.

The probability for a 40S subunit to bind a ternary complex before Factor X is *P_TC1 _*= *a_TC_*/(*a_S _*+ *a_TC _*+ *a_X_*). After acquiring ternary complex, there are only two possible reactions in the system (i.e. reactions 10 and 12). The probability of scanning changes to *P_S_*_2 _= *a_S_*/(*a_S _*+ *a_X_*), and the probability for the ribosome to traverse the remaining distance without associating with Factor X would be $P_{S2}^{n-i}$. Hence, $\left(1-P_{S2}^{n-i}\right)$ gives the probability of assembling Factor X before reaching the *GCN4 *ORF.

In summary, the probability for a 40S subunit to scan *i *times first, bind ternary complex and then Factor X before reaching position *n *is:

(16) $$P_{S1}^{i}\times P_{TC1}\times \left(1-P_{S2}^{n-i}\right)$$

Similarly, the probability of a 40S subunit to scan *i *times first, bind Factor X first and then ternary complex before reaching position *n *is:

(17) $$P_{S1}^{i}\times P_{X1}\times \left(1-P_{S3}^{n-i}\right),$$

where *P_X_*_1 _= *a_X_*/(*a_S _*+ *a_TC _*+ *a_X_*), *P_S_*3 = *a_S_*/(*a_S _*+ *a_TC_*).

Summing the probabilities of these two possible sequences of events, the probability for a ribosome to bind both factors before finishing traversing *n *nucleotides is:

(18) $$\begin{array}{c} P_{1}\left(n\right)=\\ \overset{n}{\underset{i=0}{\sum }}\left\{P_{S1}^{i}\times \left[P_{TC1}\times \left(1-P_{S2}^{n-i}\right)+P_{X1}\times \left(1-P_{S3}^{n-i}\right)\right]\right\} \end{array}$$

The case depicted in Figure 1C, in which both uORF1 and uORF4 are present in front of *GCN4 *is more intricate. Here, a ribosome has to assemble a ternary complex while scanning between uORF4 and the *GCN4 *ORF, and must bind Factor X before the *GCN4 *ORF. This is divisible into two possibilities depending on where the ribosome is when Factor X is acquired. If the ribosome binds Factor X before reaching uORF4 (after scanning *i *times), it has to reach uORF4 without ternary complex, and associate with ternary complex before the *GCN4 *ORF. Thus, the probability of *GCN4 *reinitiation in this case is:

(19) $$P_{2}\left(n_{1},n_{2}\right)=\overset{n_{1}}{\underset{i=0}{\sum }}\left[P_{s1}^{i}\times P_{X1}\times P_{S3}^{n_{1}-i}\times \left(1-P_{s3}^{n2}\right)\right].$$

Alternatively, the ribosome could bind Factor X after it scans past the uORF4 start codon. In this case, it has to bind both factors before the *GCN4 *ORF. The probability of *GCN4 *reinitiation via this sequence of events is the product of the probability of scanning past the uORF4 start codon without any factors with the probability of binding both factors afterwards:

(20) $$\begin{array}{c} P_{3}\left(n_{1},n_{2}\right)=\\ P_{S1}^{n_{1}}\times \overset{n_{2}}{\underset{i=0}{\sum }}\left\{P_{S1}^{i}\times \left[\begin{array}{c} P_{TC1}\times \left(1-P_{S2}^{n_{2}-i}\right)\\ +P_{X1}\times \left(1-P_{S3}^{n_{2}-i}\right) \end{array}\right]\right\}. \end{array}$$

In summary, the theoretical value for $P_{GCN4}^{obs}\left(n_{1},n_{2}\right)$ is

(21) $$P_{GCN4}^{theo}\left(n_{1},n_{2}\right)=P_{1}\left(n_{1}+n_{2}\right)\times 100\%,$$

and the theoretical value for $P_{uORF4}^{obs}\left(n_{1},n_{2}\right)$ is:

(22) $$\begin{array}{c} P_{uORF4}^{theo}\left(n_{1},n_{2}\right)=\\ \frac{P_{1}\left(n_{1}+n_{2}\right)-P_{2}\left(n_{1},n_{2}\right)-P_{3}\left(n_{1},n_{2}\right)}{P_{1}\left(n_{1}+n_{2}\right)}\times 100\% \end{array}.$$

### Model implementations

Having formulated the translation of uORF4 and *GCN4 *main ORF, we parameterised Model 1 using the published data graphed in Figure 3[4]. An evolutionary algorithm was employed to minimise the Euclidian Distances between experimental data and model predictions under the repressing and derepressing conditions, separately, with population size of 200 and 200 generations. The optimal values for *a_TC_/a_S _*and *a_X_*/*a_S _*are summarised in Table 1[11]. As depicted in Figure 3, Model 1 fits both conditions for uORF4 and *GCN4 *quantitatively well. In addition, parameter dependency of model fitness to the experimental data is shown on Figure S1 (Additional file 2). More details are available in the Supporting Information (Additional file 1). To run the parameter estimation of Model 1, copy the following files (Additional file 3. Model_1_parameterisation.m; Additional file 4. isres.m; Additional file 5. srsort.c) into the same folder. Mex srsort.c in MATLAB^® ^environment and run Model_1_parameterisation.m. Model 1 is available as a standalone MATLAB script (Additional file 6. Model_1.m). All models are developed in MATLAB^® ^R2007a (The MathWorks Inc.), and are available as additional files.

**Table 1:** Relative binding rates for ternary complex and Factor X compared with a fixed scanning rate (30 nt/s) under the two conditions

	Relative TC binding rate (*a_TC_*/*a_S_*, %)	Relative Factor X binding rate (*a_X_*/*a_S_*, %)
Repressing	4.6	0.72

Derepressing	0.57	5.4

## Results

### *GCN4 *translation is affected by intercistronic distances

**Figure 4:**
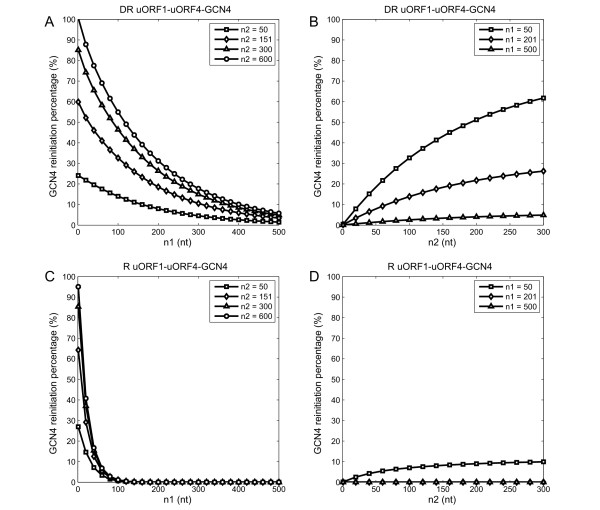
**The effect of intercistronic distances on GCN4 reinitiation (Model 1)**. This is examined by changing each distance individually (*n*_1_: A and C; *n*_2_: B and D) under the two conditions (derepressing: A and B; repressing: C and D).

Several testable predictions arise from Model 1. Firstly, decreasing *n*_1 _is expected to reduce the time taken for a ribosome to reach uORF4, thereby decreasing the chance of binding ternary complex before reaching uORF4. Hence, decreasing *n*_1 _would be expected to promote *GCN4 *translation (Figure 4C, D). In particular, for a construct as depicted in Figure 1C, if *n_1 _*is radically truncated from the natural length to less than 50 nucleotides, Model 1 predicts that the percentage of ribosomes that reinitiate at *GCN4 *would remain high under repressing conditions, as long as *n_1 _*is longer than 50 nt (see the line marked by squares in Figure 4D). Grant *et al*. [4] have shown experimentally that when *n*_1 _is reduced to only 32 nucleotides, the mutants would always exhibit high *GCN4 *expression (Table 2: compare mutant 1 with mutant 4 under repressing condition) [4]. These predictions agree quantitatively well with the results.

**Table 2:** Percentage of reinitiation at *GCN4 *as observed in experiments and predicted by Model 1

	Mutant 1		Mutant 2		Mutant 3		Mutant 4	
	
	*n*_1_	*n*_2_	*n*_1_	*n*_2_	*n*_1_	*n*_2_	*n*_1_	*n*_2_
	
*GCN4-lacZ *Mutants	32	151	32	294	200	100	200	150
DR(%) measured^a ^(predicted)	38.8 (48.4)		55.4 (68.3)		13.7 (13.9)		27.9 (18.5)	

R(%) measured^a ^(predicted)	14.1 (17.6)		30.3 (21.8)		1.23 (0.0115)		2.81 (0.0121)	

^a^Construct is depicted in Figure 1C (contains uORF4 and *GCN4*). Calculated using published data from [5] by Equation 2.

Secondly, elongating *n*_2 _(while fixing *n_1_*) would be predicted to increase the time it takes for a ribosome to scan to the *GCN4 *ORF after bypassing uORF4. This would be expected to favour binding of ternary complex and Factor X, both of which are required for *GCN4 *start codon recognition (Figure 4A, B). For instance, when *n*_1 _is reduced to only 32 nucleotides, it was found that doubling the distance *n*_2 _(from the natural distance of 151 nucleotides to 295 nucleotides) increases *GCN4 *translation activity by about 40% under derepressing conditions [4]. This experimental observation is consistent with model prediction (Table 2: compare mutant 1 with mutant 2 under derepressing conditions).

In addition, Model 1 also predicts that about half of the ribosomes that bypass uORF4 will bind both the ternary complex and Factor X after scanning 100 nucleotides downstream of uORF4. This is in accordance with the experimental observation that *GCN4 *translation is lowered by around 50% when an additional artificial uORF is inserted 100 nucleotides downstream of uORF4 (Table 2: compare mutant 3 with mutant 4 under derepressing conditions) [4].

Interestingly, the model also predicts that the natural length for *n*_2 _does not guarantee that all ribosomes that bypass uORF4 will reinitiate at *GCN4*. For example, under derepressing conditions, lengthening *n*_2 _from 150 nucleotides to 600 nucleotides is expected to lead to an increase in *GCN4 *translation of up to 70%, irrespective of the *n*_1 _value (Figure 4A: compare the line marked by diamonds with the line by circles). This is an interesting prediction that could be tested experimentally.

### *GCN4 *translation is regulated via modulation of ternary complex levels

**Figure 5:**
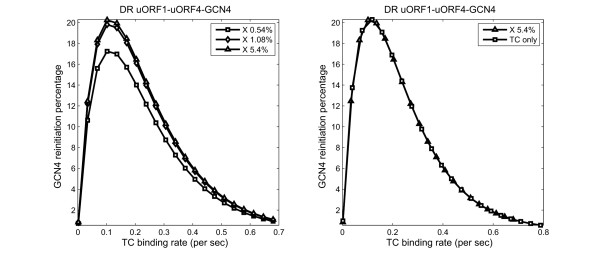
**Dependence of *GCN4 *translation on ternary complex and Factor X levels**. Percentage of ribosomes that reinitiate at *GCN4 *is computed for ternary complex levels ranging from the repressing condition to only 1% of the scanning rate (A, B). (A) Using Model 1, *GCN4 *reinitiation percentages were calculated for the normal Factor X levels (triangle) and 5-(diamond) and 10-fold reductions (square) in Factor X. (B) Model 2 (square) is parameterised and plotted on top of Model 1 (triangle).

An important facet of *GCN4 *translational regulation is its dependence on the levels of the ternary complex. We have investigated this using Model 1 that describes a *GCN4 *mRNA with uORF1, uORF4 and the main *GCN4 *ORF. Decreasing ternary complex levels is expected to impose two opposite effects on *GCN4 *translation. On one hand, a reduction in ternary complex levels favours the bypassing of uORF4 and hence increased *GCN4 *translation. On the other hand, *GCN4 *translation is inhibited when the levels of ternary complex are too low to allow the 40S subunit to bind ternary complex while it scans from uORF1 towards the main *GCN4 *ORF. Model 1 (Equation 22, $P_{GCN4}^{theo}\left(n_{1},\;n_{2}\right)$) delineates this dependency and predicts an optimum ternary complex binding rate *a_TC _*for *GCN4 *translation under derepressing conditions (Figure 5A, Table 1). A further reduction in ternary complex below this point is expected to lead to a dramatic decrease in *GCN4 *translation as the second effect becomes predominant (Figure 5A).

Similarly, we have also investigated how changes in the level of Factor X would affect *GCN4 *translation under derepressing conditions. As shown in Figure 5B, Model 1 predicts that a five-fold reduction in Factor X would have no detectable effect upon optimum *GCN4 *reinitiation, while even a ten-fold decrease in the abundance of Factor X would only lead to a reduction of roughly 20% in the optimum reinitiation frequency at the *GCN4 *ORF. The reason why *GCN4 *reinitiation appears to be relatively insensitive to Factor X concentration is that the natural uORF1-*GCN4 *distance appears to be long enough to ensure efficient Factor X binding, even when Factor X levels are significantly reduced. For a construct that contains only uORF1 and *GCN4 *(as shown in Figure 1D), all ribosomes bind Factor X and translate *GCN4 *under both repressing and derepressing conditions, as shown in Figure 3B. We conjecture that the natural *GCN4 *mRNA has probably been evolutionarily selected to minimise any undesirable effects of Factor X on *GCN4 *translational regulation. Consequently, Factor X might not have a significant impact upon the dependence of *GCN4 *translation upon ternary complex levels when uORF1 and *GCN4 *are separated by the natural distance of 350 nucleotides or more. Any distance shorter than this, like in the experiments carried out by Grant *et al*, would make the effect of Factor X more obvious (Figure 3). In summary, Model 1 reveals that the intercistronic distances *n*_1 _and *n*_2 _are critical for the regulation of *GCN4 *mRNA translation, and that the natural distances may minimise the complicated effects of Factor X.

### Model 2: The translation of naturally occurring *GCN4 *mRNA can be modelled without considering Factor X

Since we are interested in the translation of *GCN4 *mRNA with natural intercistronic distances, from now on, we neglect the existence of Factor X, and generate a simpler probabilistic model (Model 2, available in MATLAB format as Additional file 7. Model_2.m) to address how ternary complex binding rate controls *GCN4 *translation. Of course, this model would not be able to explain the data concerning *GCN4 *mutations with unnaturally short *n_2 _*distance (Figure 3B). But our imperative is to study the translational control for *GCN4 *mRNA with natural uORF1 and uORF4 placements.

In Model 2, a ribosome either scans or binds ternary complex. The probability of scanning at each step is *P_S _*= *a_S_*/(*a_S _*+ *a_TC_*). Hence, the probability of reaching uORF4 without binding ternary complex is $P_{s}^{n_{1}}.$ In other words, the probability for a ribosome to reinitiate at uORF4 is:

(23) $$P_{uORF4}^{theo}=1-P_{s}^{n_{1}}.$$

Similarly, the probability for a ribosome to reinitiate at the *GCN4 *ORF (i.e. binding ternary complex between uORF1 and *GCN4*) is:

(24) $$P_{GCN4}^{theo}=P_{s}^{n_{1}}\left(1-P_{s}^{n_{2}}\right)=P_{s}^{n_{1}}-P_{s}^{n_{1}+n_{2}}.$$

The binding rate for ternary complex relative to the scanning rate was estimated by fitting the experimental data in Figure 3A to Model 2. To parameterise Model 2, copy the following files (Additional file 4. isres.m; Additional file 5. srsort.c; Additional file 8. Model_2_parameterisation.m) into the same folder. Mex srsort.c in MATLAB and run Model_2_parameterisation.m. The resulting values (*a_TC _*relative to a constant scanning rate: repressing 4.5%; derepressing: 0.66%) were similar to those for Model 1 (compare these values with Table 1). In addition, we used Model 2 to predict the effects of ternary complex levels upon the translation of a *GCN4 *mRNA with natural intercistronic distances (Figure 1C). These predictions were then compared with those generated by Model 1. The ternary complex dependency curves for the two models were nearly superimposable, with similar optimal values for ternary complex binding rates (Figure 5B). This confirms that *GCN4 *translational regulation is essentially captured by the simplified Model 2, and that Factor X is not required to explain the translational behaviour of wild type *GCN4 *mRNA.

### Model 3: Stochastic simulation of ribosome distribution on the *GCN4 *mRNA

The first and second probabilistic models analysed the translational control of *GCN4 *mRNAs under repressing and derepressing conditions, exploiting data generated using *GCN4-lacZ *fusions. We then extended this work by constructing a third stochastic model that exploits data about ribosome loading on *GCN4 *mRNA. These data provide another important reflection of the *in vivo *translational status of this mRNA. Arava *et al*. [12] surveyed the polysome size of different sections of the *GCN4 *mRNA: a 5'-section comprising the 5'-untranslated region, and a 3'-section representing the coding and 3'-untranslated regions. They found that the 5'-section carries about one ribosome under repressing conditions, and about two ribosomes under derepressing conditions, whereas the 3'-section of the *GCN4 *mRNA has no ribosomes under repressing conditions, and about four ribosomes under derepressing conditions [12]. It is worth noting that 3-aminotriazole (3-AT) was used to induce amino acid starvation in these experiments, whereas in the above mentioned experiments that assayed *GCN4-lacZ *activity, *gcd *mutants were used to mimic derepressing conditions [4]. In *S. cerevisiae gcd *mutants, the levels of ternary complex are believed to be lowered to a similar degree to the 3-AT condition, although charged histidyl-tRNA levels are not affected in these *gcd *cells [4]. The presence of 3-AT also lowers the levels of charged histidyl-tRNA, inhibiting histidine biosynthesis [12]. Hence, the differential polysome sizes under the two conditions have allowed us to explore the effects of reducing the levels of charged histidyl-tRNA.

We were interested in correlating the observed changes in *GCN4 *ribosome loading with changes in specific kinetic parameter values (e.g. rates of initiation, scanning, and elongation, etc) under derepressing and repressing conditions. The levels of ternary complex affect the abundance of charged 43S ribosomal subunits, and hence the rate at which these 43S ribosomal subunits load onto the 5'-end of the *GCN4 *mRNA. For simplicity, we treated the ternary complex binding rate and 5'-initiation rate as two independent factors in the subsequent discussion. To distinguish the most dominant kinetic parameter values in determining *GCN4 *ribosome loading, we took the relative ternary complex binding rates from Model 2. Using MATLAB, we placed these rates in a stochastic simulation framework that describes the behaviour of ribosomes on the *GCN4 *mRNA. As depicted in Figure 2, the *GCN4 *mRNA in Model 3 contains uORF1, uORF4 and the main *GCN4 *ORF. Also, Model 3 inherits all of the simplifications defined for Model 1 and Model 2 (above). Multiple ribosomes were allowed on a single mRNA simultaneously. We assume that each ribosome occupies 36 nucleotides of mRNA, irrespective of its position in the mRNA [13]. If one scanning ribosome encountered a second ribosome, its progress would be sterically hindered. This issue was not addressed in either of the first two models. The biochemical equations that underpin Model 3 are available as additional files. To simulate Model 3, copy Additional file 9. GCN4_translation.m and Additional file 10. GCN4_codon_rate.txt into the same folder and run GCN4_translation.m in MATLAB.

Simulations were carried out using the Gillespie algorithm, starting each with an unloaded *GCN4 *mRNA. In these simulations we ensured that the system reached steady-state and that enough data were generated to generate statistically significant results. Simulations were carried out for 60 minutes, and data from 10-60 minutes were analysed to make sure that the system reached the steady state. Subsequently, we averaged the ribosome loading on each *GCN4 *mRNA over time to generate one data point for a specific condition. Fifty such generated data points were then averaged for each condition to calculate the ribosome loading. Because this system is ergodic, this method of averaging provides a view of the translation of multiple copies of *GCN4 *mRNA in many cells. Of course, we note that the half-life of the *GCN4 *mRNA is around 19 minutes under repressing conditions [14]. Using simulations to understand the noise in *GCN4 *mRNA translation due to mRNA degradation is a different issue and is outside the scope of this paper.

### 5' polysome size is determined by the ratio between 5'-initiation rate and scanning rate

Using Model 3 we first investigated the impact of individual parameters upon ribosome loading on the 5'-section of the *GCN4 *mRNA (i.e. the 5'-leader region). These parameters included the rate of translational initiation at the 5'-end of the *GCN4 *mRNA *a_I_*, the 40S/48S scanning rate *a_S _*and the rate of ternary complex binding *a_TC_*. Besides, we also considered the rate of 60S association, the translational elongation rate, and the rate of translational termination (whether the ribosome remains associated or dissociates from the mRNA following termination). These events were not considered in Models 1 and 2 for the sake of simplication. The translational elongation rate for each codon and the termination rates for *S. cerevisiae *were taken from a previous study [15]. Recent studies suggest that the rate of association of the 60S subunit is not rate limiting for translation [16]. Neither is the rate of 60S association controlled under different nutritional conditions [17]. Hence, the rate of this step was assumed to be constant and equal to the average translational elongation rate (30 nt/s). In addition, the *GCN4 *uORFs are short, containing only four codons, and consequently their translation was presumed not to be limiting. The rates of translational initiation *a_I _*ribosome scanning *a_S _*and ternary complex binding *a_TC _*were likely to be the most important parameters that influence the ribosome loading on the 5'-leader of the *GCN4 *mRNA. Therefore, we focused mainly on these parameters.

We first analysed the effects of 5'-translational initiation *a_I _*and ribosome scanning *a_S_*. The absolute rate of scanning *in vivo *is unknown, but it is expected to be at least comparable to the translational elongation rate. Therefore, we explored scanning rates within a physiologically relevant range (from 5 to 100 nt/s).

**Figure 6:**
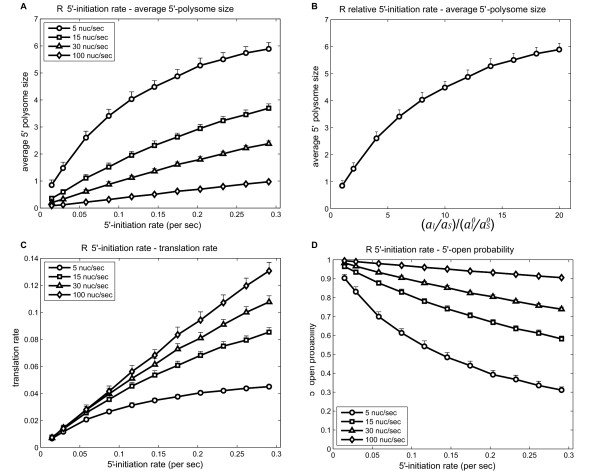
**5'-initiation rate and scanning rate impact on 5'-UTR translation under repressing conditions (Model 3)**. (A) For constant 60S subunit joining (30 nt/s), translation elongation and termination rates (calculated by Gilchrist and Wagner, [15]), the average 5'-polysome sizes were calculated for different scanning rates (5, 15, 30, 100 nt/s) under different 5'-initiation rates (from 0.0145 to 0.29 s^-1^). The repressing ternary complex binding rate was predicted by the simplified model. *GCN4 *mRNA translation is simulated for 60 minutes, and the results are averaged over the time period of 10-60 min. Each point represents an average of 50 such replicates. And the error bars denote 1 standard deviation. Some error bars are too short to be seen. Data in C and D were averaged in the same way. (B) Average 5'-polysome size was plotted against the ratio *a_I_*/*a_S _*relative to its nominal value. Simulation results at scanning rate of 5 nt/s from A were used. (C) uORF1 translation rate is calculated for different 5'-initiation rates at each scanning rate. (D) The probability that the 5'-end is unoccupied and available to receive an initiating ribosome is calculated for different 5'-initiation rates at different scanning rates.

Due to the steric hindrance, ribosome loading on the *GCN4 *5'-leader tends to become saturated as the rate of translation initiation *a_I _*at the 5'-end of the mRNA increases, as shown in Figure 6A. Under repressing conditions, at the scanning speed of 5 nt/s, a six-fold increase in 5'-initiation rate (from 0.0145 to 0.0870) leads to about a three-fold increase in ribosome loading on the *GCN4 *5'-leader region. Steric hindrance has most pronounced effects when scanning rate is limiting (Figure 6A: 5 nt/s curve). At higher scanning speeds, the saturation trend is reduced but still visible (Figure 6A: curves at 15 and 30 nt/s scanning speeds). When scanning speed is very high, 5' polysome size increases approximately linearly with translation initiation rate *a_I _*(Figure 6A: curve at 100 nt/s scanning speed).

**Figure 7:**
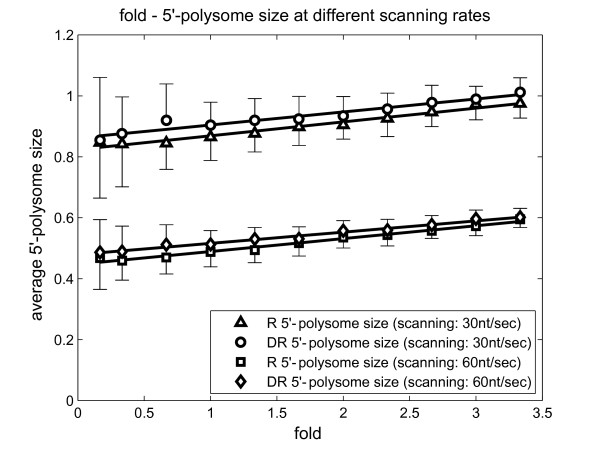
**The impact of absolute 5'-initiation rate and scanning rate on 5'-polysome size (Model 3)**. 5'-initiation rate ' is changed proportionally with scanning rate (from 5 nt/s to 100 nt/s), and maintains a constant ratio such that the relative 5'-initiation rate is 1. *Fold *= *a_S _*/30. The other rates, including those of 60S subunit joining (30 nt/s), translation elongation and termination (calculated by Gilchrist and Wagner, [15]) were kept constant. Ternary complex binding rates *a_TC _*under the two conditions predicted by Model 2 were used to generate the simulations. Each data point represents an average of 50 replicates, in the same way as in Figure 6.

It is interesting to note that the 5' polysome size is mainly determined by the ratio *a_I_*/*a_S_*, irrespective of the fact whether scanning is limiting or not. In Figure 7, the polysome sizes at (*a_I _*= 0.029*s*^-1^, *a_S _*= 5 nt/s), (*a_I _*= 0.087*s*^-1^, *a_S _*= 15 nt/s) and (*a_I _*= 0.174*s*^-1^, *a_S _*= 30 nt/s) are approximately identical. Similarly, polysome sizes at (*a_I _*= 0.087*s*^-1^, *a_S _*= 5 nt/s) and (*a_I _*= 0.174*s*^-1^, *a_S _*= 15 nt/s) are the same, so on and so forth.

We further tested this idea. At scanning speed of 30 nt/s, we increased the absolute rates of 5'-initiation *a_I _*and scanning *a_S _*proportionately, and observed how 5' polysome size changed (Figure 7). Model 3 predicted that there will only be a slight increase in the ribosome loading on the *GCN4 *5'-leader (of about 5%) if these two rates are simultaneously elevated together over a five-fold range, under both repressing and derepressing conditions (Figure 7). If the ratio between the rates of 5'-initiation *a_I _*and scanning *a_S _*was changed by doubling the scanning rate from 30 to 60 nt/s, then this was predicted to lead to a two-fold decrease in the ribosome loading on the *GCN4 *5'-leader. These results reinforce the view that 5' polysome size is largely dependent on the ratio between the 5'-initiation *a_I _*and scanning rate *a_S_*. Figure 7 shows how this ratio changes 5' polysome size. These results can be explained by the theoretical results from the totally asymmetric exclusion progress [18,19].

Arava and colleagues found that when cells grown in rich media undergo amino acid starvation, the ribosome loading in the 5'-section of the mRNA changes from 1 to 2. The ratio *a_I_*⁄*a_S _*is suggested to increase three-fold upon this change (Additional file 11. Figure S2). This might be due to a three-fold increase in *a_I_*, or a three-fold decrease in *a_S_*. It is also likely that both parameters may change simultaneously under derepressing conditions. This is discussed in detail in a later subsection.

### 5' polysome size is not significantly affected by the ternary complex binding rate *a_TC_*

We also investigated how ternary complex binding rate *a_TC _*affects ribosome loading in the 5'-section. Comparing Figure 6A (repressing) with Figure S2 A (derepressing, Additional file 11), it is apparent that this effect is negligible. Changes in *a_TC _*itself would only affect the probability for a 40S ribosomal subunit to bypass uORF4 and hence change the ribosome density in the section between uORF4 and *GCN4 *ORF. However, *n_2 _*is only about 1/4 of the entire 5'-section. Consistent with Model 1, only about 25% of the 40S subunits downstream of uORF1 would bypass uORF4 under derepressing condition (Figure 3A). Taken these two factors together, ternary complex binding rate *a_TC _*does not significantly influence 5' polysome size. This is reinforced by the observation in Figure 7, where the 5' polysome size stays approximately the same under the two conditions.

### Rates of 5' translation initiation *a_I _*and scanning *a_S _*affect other aspects of translation

Besides 5' polysome size, *a_I _*and *a_S _*affect other aspects of *GCN4 *mRNA translation. We found that when the rate of scanning is limiting, uORF1 translation becomes solely dependent on this parameter. In this case, a higher translation rate is not achievable through increases in the rate of translation initiation at the 5'-end of the mRNA. Hence, the rate of uORF1 translation tends to become saturated as 5'-initiation rates increase, and this trend is most pronounced at low scanning rates (Figure 6B: the curve for 5 nt/s). When scanning is not limiting, uORF1 translation rate is almost linearly proportional to the 5'-initiation rate (Figure 6B: the curve for 100 nt/s). In addition, at slow scanning rates, it takes a relatively long time for a ribosome to move from the first 36 nucleotides of the *GCN4 *mRNA to expose the 5'-end and allow another round of translational initiation. Hence, at slower scanning rates the 5'-end is less likely to be unoccupied (Figure 6C: please compare the curves for 5 and 15 nt/s). Similarly, at high translational initiation rates the 5'-end of the mRNA is more likely to be occupied.

### Ternary complex binding rate affects ribosome loading on the 3'-section of the *GCN4 *mRNA

Next, we investigated how different parameters are predicted to change the ribosome loading in the 3'-section of the *GCN4 *mRNA (Figure 2), and related these to the experimental data on 3'-polysome size [12]. Intuitively, the rates of 5'-initiation, ternary complex binding, and histidine codon translation in the *GCN4 *ORF would be expected to alter ribosome loading in the 3'-section of the *GCN4 *mRNA. Hence, it is not possible to adjust the 5'-initiation rate *a_I _*alone to satisfy the experimental data on ribosome loading in both the 3'-and 5'-sections of the mRNA simultaneously, because the values of *a_TC _*and histidine codon translation rates are unknown. As mentioned previously, the ratio *a_I_*/*a_S _*is suggested to increase from one to about three when cells change from repressing to derepressing conditions. We started by investigating two extreme cases that correspond to two ribosomes in the 5'-section of the *GCN4 *mRNA: firstly a three-fold decrease in scanning rate (to 10 nt/s, nominal value at 30 nt/s); and secondly a three-fold increase in 5'-initiation rate (to 0.26 *s*^-1^, nominal value at 0.087 *s*^-1^). This transformed the problem into whether values could be found for the rates of ternary complex binding and histidine codon translation that allow four ribosomes in the 3'-section of the mRNA under 3-AT conditions.

**Figure 8:**
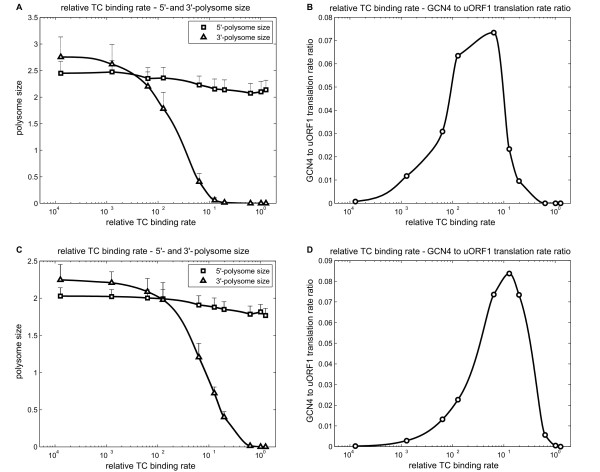
**Effects of ternary complex binding rate (Model 3)**. This effect was analysed under circumstances where scanning rate was decreased by 3-fold (A and B), and 5'-initiation rate was increased to 3-fold (C and D). *GCN4 *mRNA translation was simulated at each specific ternary complex binding rate for 60 min. The polysome sizes for the two sections were averaged over 10-60 min. 50 such replicates were averaged to determine the polysome sizes (A and C) for each ternary complex binding rate. The translation rate of uORF1 and *GCN4 *was also monitored. Subsequently, we calculated the ratio between *GCN4 *translation and uORF1 translation for each ternary complex binding rate in the same way of averaging (B and D).

Ternary complex binding rate was first investigated. We asked if changes in ternary complex alone were sufficient to explain the changes in the 3'-ribosome loading under the two conditions, and explored the effect of ternary complex levels on Gcn4 protein production when other parameters were kept constant. Our simulations predicted that the 3'-ribosome loading is significantly less than one under the optimised derepressing conditions revealed by Model 2 (i.e. *a_TC_/a_S _*= 4.5%) (Figure 8A and 8C). In fact, in each case, Model 3 predicted that the highest 3'-ribosome loading is about 10% higher than the 5'-ribosome loading (Figure 8A and C). This is inconsistent with the experimental observation that the 3'-ribosome loading was around four under amino acid starvation (derepressing) conditions. As shown in Figure 8A and 8C, when the ribosome loading is roughly equal on each section of the *GCN4 *mRNA, ternary complex binding rate is decreased by about 100-fold. The ratio of the translation rates for the *GCN4 *ORF to uORF1 provides a gauge of *GCN4 *translational status. Figure 8B and 8D demonstrate that *GCN4 *to uORF1 translation rate ratio is only 20-40% of the optimal level under these circumstances. These two observations suggest that changes in ternary complex binding rate alone are unable to account for the experimental data. Other parameters appear to play roles in determining the ribosome loading on the 5'-and 3'-sections of the *GCN4 *mRNA. We investigated the effects of changing the rate of translation elongation of histidine codons.

### The rate of translational elongation on histidine codons influences the ratio of ribosome loading on the 3'-and 5'-sections of the *GCN4 *mRNA

**Figure 9:**
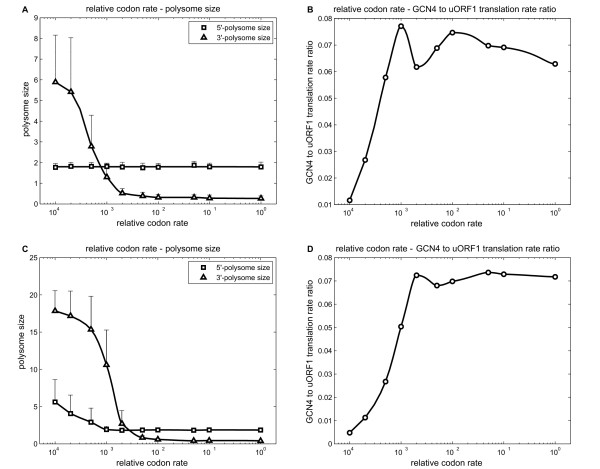
**Effects of histidine codons translation rates (Model 3)**. This effect was analysed under circumstances where scanning rate was decreased by 3-fold (A and B), and 5'-initiation rate was increased to 3-fold (C and D). To ensure a constant *GCN4 *reinitiation rate, Ternary complex binding rate was set to maintain its derepressing ratio with the scanning rate under each condition (10 and 30 nt/s, respectively). Other parameters were the same as in Figure 6. The two histidine codon rates were changed to the same degree, from 0.0001-to 1-fold relative to its repressing value (i.e. relative codon rate). Simulations results were obtained and averaged in the same way as Figure 8. 5'-and 3'-polysome sizes (A, C) and uORF1 to *GCN4 *translation rate ratio (B, D) were plotted against relative codon rate.

The *GCN4 *mRNA has four histidine codons, all of which are present in the main *GCN4 *ORF (Figure 2). To analyse the effects of decreasing the concentration of histidyl-charged tRNA under amino acid starvation conditions, replicated using the competitive inhibitor of histidine biosynthesis, 3-AT, we investigated the effects of varying the rate of translation of histidine codons. In these simulations the rates of translation of the two types of histidine codon (CAU and CAC) were varied to the same degree (from 0.0001-fold to 1-fold of the rate under repressing conditions). We first analysed the case of 3-fold reduction in ribosome scanning rate, monitoring the impact upon ribosome loading in both sections of the *GCN4 *mRNA. To retain the same probability of *GCN4 *reinitiation in these simulations, the ternary complex binding rate was also lowered three-fold (Figure 9A and 9B). As shown in Figure 9A, the decrease in histidine codon translation rate was not predicted to affect 5'-ribosome loading. However, 3'-ribosome loading increased to a value of four when histidine codon translation rates were reduced about 0.0004-fold, for example by 3-AT. At the same time, the relative translation rate of the *GCN4 *ORF does not change significantly when histidine codon rates are reduced to 0.0004-fold (Figure 9B). This suggests that slow histidine codon translation rates contribute to the increase in 3'-ribosome loading, yet do not impede *GCN4 *translation.

To test this idea further, we then examined the effects of a three-fold increase in 5'-initiation activity. Figure 9C shows that when histidine codon translation rates are decreased around 0.002-fold, the 3'-ribosome loading was roughly four. In addition, the relative translational activity of the main *GCN4 *ORF is not impeded relative to the uORF1 translation rate (Figure 9D). This lends weight to the hypothesis that a decrease in the rate of translation of histidine codons causes a significant increase in ribosome loading on the *GCN4 *ORF during histidine starvation (treatment with 3-AT).

Ribosome loading on an mRNA may increase due to a limiting translational termination rate. This might also account for an increase in 3'-ribosome loading. Thus, we analysed the impact of translational termination upon ribosome loading on the *GCN4 *mRNA. Model 3 predicts that, under derepressing conditions, when translational termination becomes limiting, 5'-ribosome loading increases whilst 3'-ribosome loading decreases essentially to zero (data not shown). This is because, under these circumstances, ribosomes become restricted to the 5'-proximal region of the *GCN4 *mRNA before the uORF1 stop codon while they wait for translational termination. Hence, relatively few ribosomes are able to move beyond uORF1 to reach the *GCN4 *ORF. This in turn would lead to a significant decrease in 3'-ribosome loading. In conclusion, a limiting translational termination rate does not appear to account for the observed increase in 3'-ribosome loading under derepressing conditions.

## Discussion

### Factor X identity

Our modelling has provided insights into the identity of the cryptic Factor X, which was predicted to be one of the eukaryotic initiation factors involved in start codon selection, such as eIF1 or eIF5 [4]. Factor X is needed for *GCN4 *start codon selection, but is dispensable for uORF4 reinitiation. Model 1 predicts that the rate of Factor X binding increases under derepressing conditions. This could be explained either by an increase in eIF levels or by an increase in their rates of association with the translation initiation complex under derepressing conditions. However, the absolute abundance of eIFs does not change under repressing and derepressing conditions [20]. This prompted us to investigate the second possibility. A recent study suggests that eIF's bind the 40S ribosomal subunit cooperatively, such that the binding of one factor enhances the affinity of the initiation complex for other factors [9,21,22]. In addition, an intermediate eIF· eIF· eIF5 complex may be important for TC recruitment [23,24]. Our model of general mRNA translation in yeast suggests that the level of eIF1· eIF3· eIF5 complex increases about 20-fold during histidine starvation (derepressing conditions) [25]. It is likely that this complex binds the 40S subunit faster than the individual eIFs, thereby mimicking an increased eIF5 level that was proposed by Grant and coworkers [4]. Hence, an increase in the level of the eIF1· eIF3· eIF5 complex formation, and the subsequent enhancement of eIF association with the 40S subunit, might explain the impact of Factor X upon translation.

For the sake of parameter identifiability, the binding of TC was assumed to be independent of Factor X in Model 1. Nevertheless, it would be interesting to investigate the cooperative effects using the model. In addition, if Factor X is indeed the eIF1· eIF3· eIF5 complex, this assumption will not hold due to the cooperation in factor binding. Including such cooperative effects will perhaps affect the quantitative predictions to certain degree but will not change the results qualitatively. On the other hand, a recent study from the Asano group suggests that Factor X might be an mRNA helicase such as Ded1 or Dhp1 [26]. If this is the case, its binding to the ribosome (or mRNA ahead of it) can be considered as independent of TC binding.

### *GCN4 *Regulation by ternary complex

Models 1 and 2 suggested that a decrease in ternary complex levels leads to a gradual increase in *GCN4 *mRNA translation (Figure 5). In other words, the dependence of *GCN4 *translation upon ternary complex levels reflects analogue-type behaviour rather than an on-off switch. The structure of equations 22 and 23 in Model 2 clearly demonstrates that this relationship is endowed by nature of the stochastic regulation and is independent of kinetic parameter values. The ternary complex binding rate under the derepressing conditions that we extracted from published experimental data [4] was estimated to be 0.168 nt/s (*i.e*. 0.56% of 30 nt/s; Table 1). This was close to the optimal ternary complex binding rate for *GCN4 *mRNA derepression (Figure 5). However, the 3-AT condition under which Grant and colleagues performed their experiments could be viewed as artificial in that it caused more severe amino acid starvation than natural starvation conditions, leading to lower ternary complex levels than for natural starvation. Hence, translation of *GCN4 *mRNA operates at much higher ternary complex levels in response to natural amino acid starvations, where the relationship between the two is more linear (e.g. the region between 0.5 to 0.7 s^-1 ^in Figure 5A and 5B). Such dependence of *GCN4 *translation on ternary complex levels is perhaps advantageous. On one hand, *GCN4 *is a master transcription factor that remodels nearly a quarter of gene expression in yeast [1]. Such a linear relationship at relatively high ternary complex levels allows incremental increases in *GCN4 *expression in response to natural starvation, without generating a disproportionate amount of such potent factor. On the other hand, it also allows the cell to mount a higher degree of *GCN4 *derepression in response to more severe conditions such as 3-AT treatment.

### *In vivo *translational status of *GCN4 *mRNA

Our modelling has also provided insights into the observed increase in ribosome loading that occurs on the *GCN4 *mRNA following amino acid starvation. The existing experimental data are unable to distinguish whether this increase in ribosome loading is due to a higher 5'-initiation rate or to a decrease in ribosome scanning [12]. However, these two conditions would have different outcomes in terms of absolute Gcn4 protein production rates (i.e. the higher 5'-initiation rate has roughly 3-fold higher effect than the lower ribosome scanning). In their study of the relationship between intercistronic distance and *GCN4 *translation, Grant *et al*. [4] inactivated all uORFs preceding the main *GCN4 *ORF by point mutation and measured the activity of the *GCN4-lacZ *constructs in both *gcn *and *gcd *mutants. Under derepressing conditions, the *GCN4-lacZ *activities were roughly the same in *gcn *cells (where *GCN4 *translation is constitutively repressed) and in *gcd *mutants (where *GCN4 *translation is constitutively derepressed). Their data indicate that the rates of 5'-initiation are comparable in *gcn *and *gcd *cells [4]. However, the histidine analogue 3-AT elicits more severe amino acid starvation than is mimicked by *gcd *mutations. This is because, in addition to reducing ternary complex levels (like *gcd *mutations), 3-AT also reduces the levels of charged histidyl-tRNA by inhibiting histidine biosynthesis. Consistently, 3-AT is known to generate a strong protein synthesis defect, as reported in a recent study by Asano's group [27]. Yet, without experimental evidence, we cannot rule out a possible change in 5'-initiation rates during amino acid starvation. To meet this challenge, we require systematic assays of ribosome density combined with measurements of *GCN4 *translation rates.

Genome-wide analyses of ribosome densities have become possible through the combination of deep RNA sequencing technologies and ribosome profiling [28]. This powerful technology, which is capable of mapping ribosomes on mRNAs with single codon resolution, has provided direct confirmation of the translation of the uORFs in the *GCN4 *mRNA as well as the translational up-regulation of the main *GCN4 *ORF following amino acid starvation. Unexpectedly, increased translation of the *GCN4 *5'-leader region was also observed under these conditions [28] suggesting that additional aspects of *GCN4 *translational regulation remain to be elucidated. While our models do not reflect these as yet uncharacterised aspects of *GCN4 *translation, they have provided new insights into *GCN4 *translational regulation. Furthermore, while not all uORF-containing mRNAs are regulated using the same mechanisms as *GCN4 *[29-31], our models provide a useful platform for predictive studies on the translational regulation of other uORF-containing mRNAs.

## Conclusions

In summary, a diversity of modelling platforms was used in this study to probe the principles governing control of *GCN4 *at the translational level, and to probe the contributions made by different soluble translation factors to the control mechanism. The predictions of the models employed were validated by comparison with experimental data, and all reproduced the dependence of *GCN4 *translation on varying ternary complex levels, a crucial feature of *GCN4 *regulation.

Overall, the study revealed that the natural intercistronic distances in the *GCN4 *mRNA are sufficiently long to allow a scanning ribosome to acquire Factor X even when the levels of this factor are low. This suggested that Factor X is largely not a relevant factor in the translational regulation of *GCN4 *with natural intercistronic distances. Deployment of a stochastic model with awareness of steric interactions between ribosomes (queuing effects) revealed that changes in histidine codon translation rate, rather than alterations in ternary complex acquisition, was the key factor governing increases in ribosome loading on the *GCN4 *ORF under amino acid starvation conditions. Thus, via mathematical modelling and simulation, we have revealed novel features of *GCN4 *regulation, an important paradigm of eukaryotic translational control.

## Competing interests

The authors declare that they have no competing interests.

## Authors' contributions

TY conceived the models, developed MATLAB programmes, analyzed the data, and drafted the paper. IS participated in paper writing. MCR helped derive the simple model that eventually led to Model 1. AJPB conceived the research, supervised data analysis and participated in paper writing. GMC conceived the research, supervised data analysis and participated in paper writing. All authors read and approved the final manuscript.

## Supplementary Material

Additional file 1 **Supporting Information**. This document contains legends of Figure S1 and Figure S2; S0. Model 1 hypotheses; S1. The biochemical equations that underpin Model 3. Click here for file

Additional file 2 **Figure S1. Parameter dependency of model fitness to the experimental data**. To demonstrate this, we have defined relative binding rates as the absolute binding rate divided by the optimal binding rate for both TC and factor X. Then we varied the relative binding rates from 0.1-to-10-fold, and calculated the Euclidian Distance between model prediction and experimental data under repressing and derepressing conditions, respectively. The side views show that under repressing condition, there is a unique pair of values to optimally model experimental results. In other words, the experimental data are sufficient to uniquely identify the two binding rates. This is also true for TC binding rate under derepressing condition. However, factor X binding rate does not have a unique value under this condition. Rather, any value higher than 0.4-fold of the optimal value would approximate the experimental data equally well. This ambiguity suggests insufficient data to determine the exact value of factor X binding rate. To overcome this difficulty, we would like to suggest use more experimental value that are sensitive to factor X binding rate to determine its value. The inaccuracy in this value may affect model predictions for derepressing condition, for example, the *GCN4 *reinitiation dependency on *n*2. Nevertheless, it does not violate the conclusion that factor X binding rate is higher under derepressing condition (0.4-fold of the optimal value under derepressing condition is still more than 3-fold higher than the repressing condition value). Click here for file

Additional file 3 **Model_1_parameterisation.m**. This is a MATLAB programme for parameterisation of Model 1. Additional files 4 and 5 should be placed under the same category to run this programme. Click here for file

Additional file 4 **isres.m**. This is a MATLAB implementation of the evolutionary algorithm that is employed to parameterise Model 1 and Model 2. Click here for file

Additional file 5 **srsort.c**. This C programme provides a stochastically ranking procedure for the evolutionary algorithm implemented by Additional file 4. Click here for file

Additional file 6 **Model_1.m**. This is a MATLAB implementation of Model 1. Click here for file

Additional file 7 **Model_2.m**. This is a MATLAB implementation of Model 2. Click here for file

Additional file 8 **Model_2_parameterisation.m**. This is a MATLAB programme for parameterisation of Model 2. Additional files 4 and 5 should be placed under the same category to run this programme. Click here for file

Additional file 9 **GCN4_translation.m**. This is a MATLAB implementation of Model 3. Additional file 7 GCN4_codon_rate.txt needs to be placed under the same category to run this file. Click here for file

Additional file 10 **GCN4_codon_rate.txt**. This text file contains the translation elongation rate for each codon. It is needed to run additional file 6. This file should be placed under the same category when Model 3 is simulated. Click here for file

Additional file 11 **Figure S2. The impacts of 5'-initiation rate and scanning rate on 5'-UTR translation under derepressing conditions**. (A) For constant 60S subunit joining (30 nt/sec), translation elongation and termination rates (obtained from Gilchrist and Wagner, [15]), the average 5'-polysome sizes were calculated for different scanning rates (5, 15, 30, 100 nt/sec) under different 5'-initiation rates (from 5/344 to 100/344 s-1). The derepressing TC binding rate was predicted by the simplified model. *GCN4 *mRNA translation is simulated for 60 minutes, and the results are averaged over the time period of 10-60 min. Each point represents an average of 50 such replicates. And the error bars denote 1 standard deviation. Some error bars are too short to be seen. Data in C and D were averaged in the same way. (B) Average 5'-polysome size was plotted against the ratio *a_I_*/*a_S _*relative to its nominal value. Simulation results at scanning rate of 5 nt/sec from A were used. (C) uORF1 translation rate is calculated for different 5'-initiation rates at each scanning rate. (D) The probability that the 5'-end is unoccupied and available to receive an initiating ribosome is calculated for different 5'-initiation rates at different scanning rates. Click here for file
