# Supplementary material for: Analysing GCN4 translational control in yeast by stochastic chemical kinetics modelling and simulation
Source: BMC Syst Biol. 2011 Aug 18;5:131. doi: 10.1186/1752-0509-5-131 (PMC3201031; doi:10.1186/1752-0509-5-131)
Supplement: Additional file 1 — Supporting Information. This document contains legends of Figure S1 and Figure S2; S0. Model 1 hypotheses; S1. The biochemical equations that underpin Model 3. [file 1752-0509-5-131-S1.PDF]

SUPPORTING INFORMATION

Analysing *GCN4* Translational Control in Yeast by Stochastic Chemical Kinetics Modelling and Simulation

Tao You, Ian Stansfield, M Carmen Romano, Alistair J P Brown and George M Coghill

Contents

Figure S1. Parameter dependency of model fitness to the experimental data.

Figure S2. The impacts of 5'-initiation rate and scanning rate on 5'-UTR translation under derepressing condition.

S0. Model 1 hypotheses

S1. The biochemical equations that underpin Model 3.

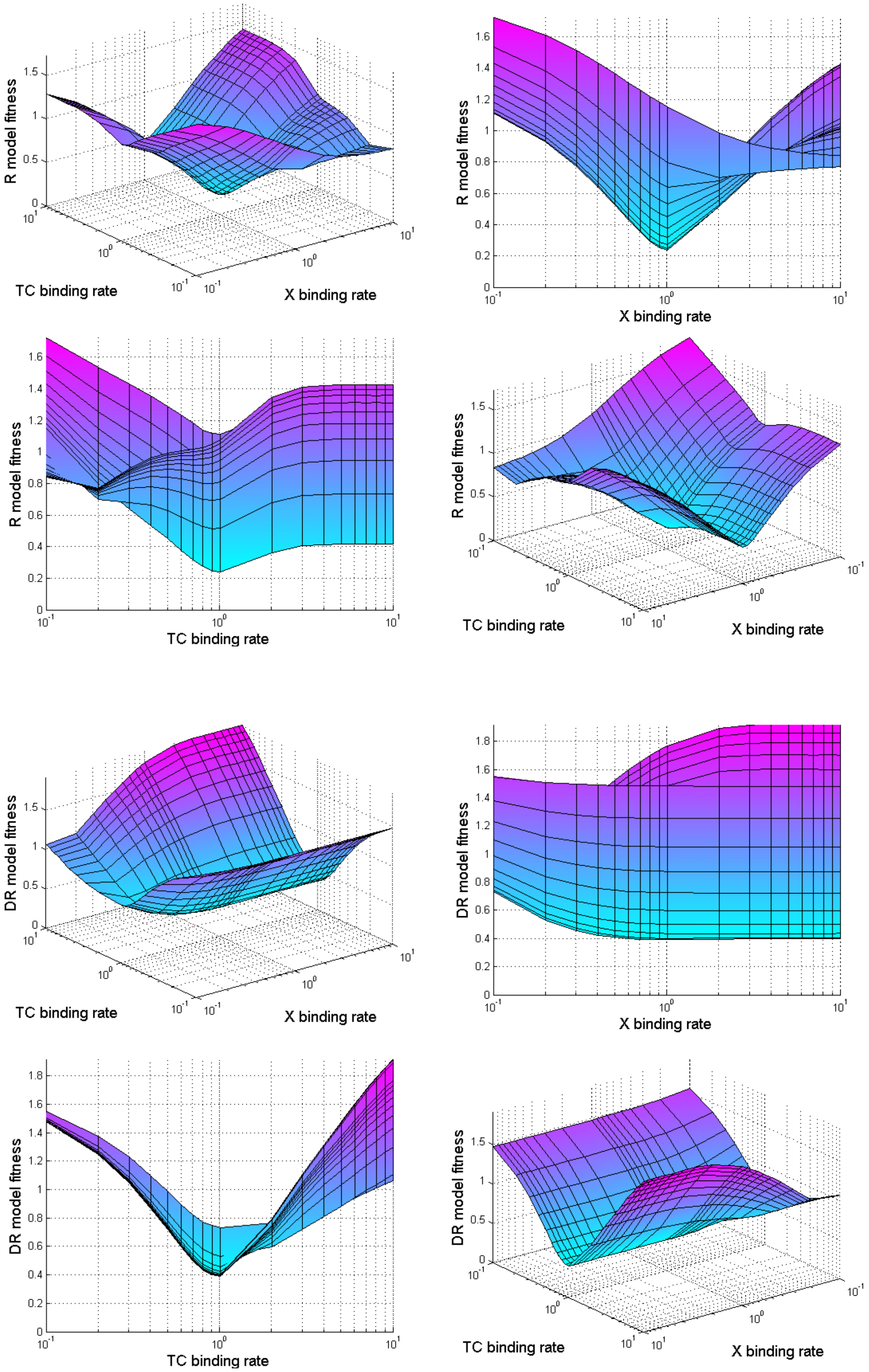

Figure S1. Parameter dependency of model fitness to the experimental data. To demonstrate this, we have defined relative binding rates as the absolute binding rate divided by the optimal binding rate for both TC and factor X. Then we varied the relative binding

rates from 0.1-to-10-fold, and calculated the Euclidian Distance between model prediction and experimental data under repressing and derepressing conditions, respectively. The side views show that under repressing condition, there is a unique pair of values to optimally model experimental results. In other words, the experimental data are sufficient to uniquely identify the two binding rates. This is also true for TC binding rate under derepressing condition. However, factor X binding rate does not have a unique value under this condition. Rather, any value higher than 0.4-fold of the optimal value would approximate the experimental data equally well. This ambiguity suggests insufficient data to determine the exact value of factor X binding rate. To overcome this difficulty, we would like to suggest use more experimental value that are sensitive to factor X binding rate to determine its value. The inaccuracy in this value may affect model predictions for derepressing condition, for example, the *GCN4* reinitiation dependency on n_2. Nevertheless, it does not violate the conclusion that factor X binding rate is higher under derepressing condition (0.4-fold of the optimal value under derepressing condition is still more than 3-fold higher than the repressing condition value).

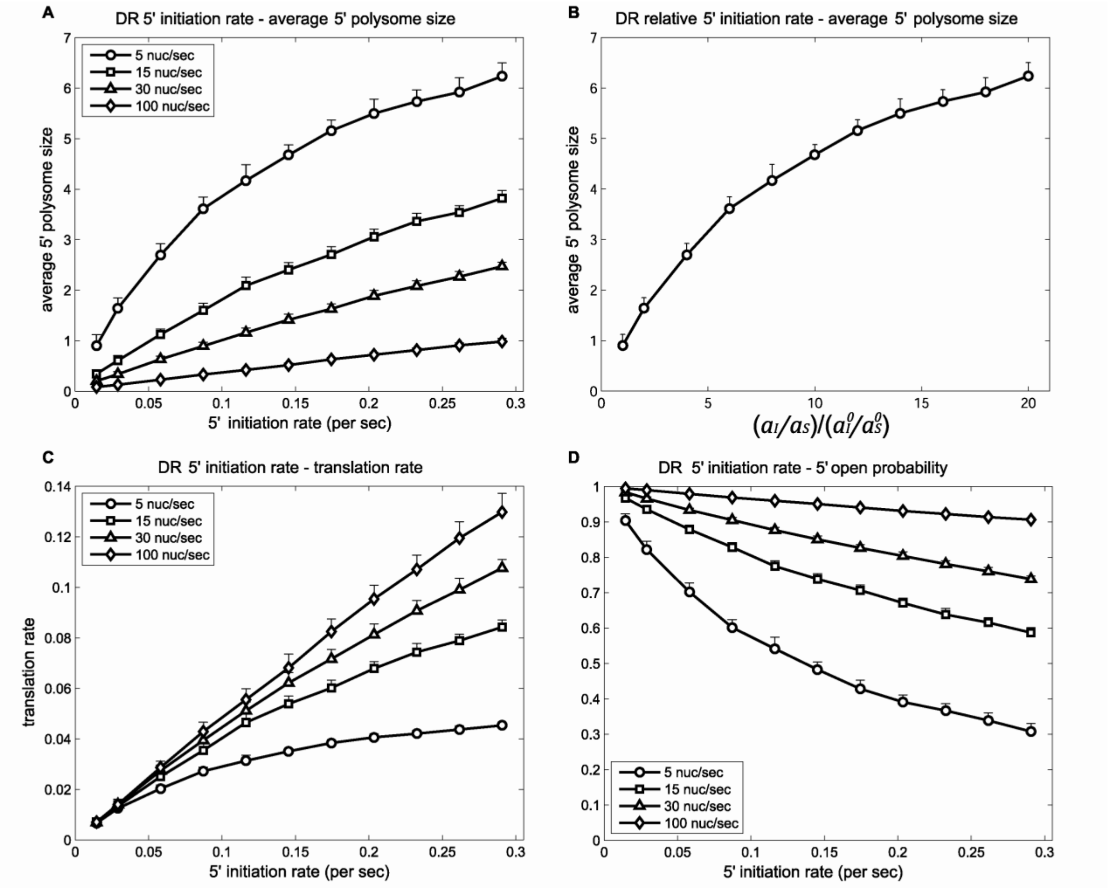

Figure S2. The impacts of 5'-initiation rate and scanning rate on 5'-UTR translation under derepressing condition. (A) For constant 60S subunit joining (30 nt/sec), translation elongation and termination rates (obtained from Gilchrist and Wagner, (15)), the average 5'-polysome sizes were calculated for different scanning rates (5, 15, 30, 100 nt/sec) under different 5'-initiation rates (from 5/344 to 100/344 s^{-1}). The derepressing TC binding rate was predicted by the simplified model. *GCN4* mRNA translation is simulated for 60 minutes, and the results are averaged over the time period of 10-60 min. Each point represents an average of 50 such replicates. And the error bars denote 1 standard deviation. Some error bars are too short to be seen. Data in C and D were averaged in the same way. (B) Average 5'-polysome size was plotted against the ratio a_I/a_S relative to its nominal value. Simulation results at scanning rate of 5 nt/sec from A were used. (C) uORF1 translation rate is calculated for different 5'-initiation rates at each scanning rate. (D) The probability that the 5'-end is unoccupied and available to receive an initiating ribosome is calculated for different 5'-initiation rates at different scanning rates.

S0. Model 1 hypotheses

Several simplifications were made to construct Model 1.

Firstly, experimental work demonstrated that *GCN4* mutants lacking uORF2 and uORF3 displayed essentially normal *GCN4* translational behaviour (2). This is because those ribosomes blocked by uORF2 and uORF3 would be blocked instead by uORF4, if those two uORFs were removed. For the sake of simplicity, only uORF1 and uORF4 are considered in the models in this paper.

Secondly, reverse scanning was not considered in the model. A recent study exploiting *in vitro* translation data was not able to exclude the possibility of reverse scanning under their experimental conditions (6). However, Kozak has demonstrated that reverse scanning has negligible effects upon start codon selection (7). Hence, we have not included reverse scanning in this model.

Thirdly, we assumed that ribosomes do not abort scanning. Ribosome scanning is a highly efficient process (6) (8). Comparisons of mRNAs with different lengths of 5'-untranslated region (from 50 to 1500 nucleotides) concluded that translational activity was not decreased by longer scanning distances (6). This implies that the random disassociation of scanning ribosomes from an mRNA is negligible.

The fourth simplification was that the ribosome scans at a constant rate. The rate of scanning has been measured indirectly to be 10 nt/sec under *in vitro* translation conditions (6). The *in vivo* scanning rate is expected to be at least the same as the rate of translation elongation (30 nt/sec) (6). In this model, we chose not to use a specific value for the scanning rate. Instead, as indicated later, we argue that ribosome reinitiation probabilities are dependent upon the ratios between scanning rates and other reactions. We have assumed that the ribosome scans at a constant speed and have investigated the relative rates of other reactions. It is worth noting that the dimension of ribosomal scanning rate is distance/time (nt/sec). For an mRNA with known length, by considering the time it takes for a ribosome to traverse the

entire mRNA, the scanning rate can be converted into molecule/sec which is directly comparable with other reaction rates. All reactions, including scanning, can thus be expressed using a common dimension, for which we chose nt/sec in this work.

Our fifth simplification was that a bound ternary complex does not dissociate from the 40S ribosomal subunit during scanning. This simplification was based on the recent observation that the dissociation of ternary complex from the 40S ribosomal subunit *in vitro* is a slow process (9). The time taken for the 40S subunit to scan the ~200 nucleotides between uORF1 and uORF4 (at 30 nucleotides per second) is about 7 seconds. This is considerably shorter than the time required for ternary complex dissociation.

Finally, we assumed that "leaky scanning" does not happen (i.e. a 43S complex recognises and translates the immediately next ORF it encounters), and that all ribosomes dissociate from the mRNA after translating uORF4. These simplifications were used previously by Grant *et al.* (4).

S0. The biochemical equations that underpin Model 3.

In Model 3, a ribosome could present in three different forms:

1. 40S ribosomal subunit free of TC at nucleotide i, denoted by 40S(i). This represents a scanning ribosome downstream of uORF1.
2. TC bound ribosome at nucleotide i, denoted by 40S·TC(i). This includes a 43S ribosomal subunit loaded onto the 5'-end of the mRNA and a scanning 40S that reacquires TC downstream of uORF1.
3. A translating ribosome at nucleotide i, denoted by 80S(i).
4. Ribosome terminated at nucleotide i, denoted by Rt(i) ("ribosome termination"). This includes termination after a ribosome translates uORF1 or uORF4 or *GCN4*, or termination when a scanning 40S (with or without TC) reaches the 3'-end of *GCN4* mRNA.

There are altogether seven possible biochemical reactions in the system as tabulated as follows.

	Reaction	Equation	Description	Condition	Nominal Rate (nt/sec)
1	Ribosomal initiation at the 5' end	→ 40S·TC(1)	43S ribosomal subunit at nucleotide 1	If there is no ribosome in the systems or if the first 36 nucleotides	30/344

				of *GCN4* mRNA are unoccupied.	
2	Rebinding TC	40S(i) → 40S·TC(i)	Reacquisition of ternary complex after uORF1 translation.	i ≥ 239	Repressing: 1.2710<br>Derepressing: 0.19712
3	Scanning	40S(i) → 40S(i+1)<br>40S·TC(i) → 40S·TC(i+1)	A non-terminated ribosome species moves by 1 nucleotide towards 3'-end	The i+1 nucleotide is unoccupied	30
4	60S ribosomal subunit joining	40S·TC(i) → 80S(i)	60S ribosomal subunit joining at uORF1, uORF4 or *GCN4*	If the ribosome is assembled with ternary complex, it recognises the immediately next start codon it encounters – no leaky scanning.<br>i = 230, 440, 591.	30
5	Ribosomal termination with dissociation	80S(i) → Rt(i) + translation product	Translation product could be polypeptide encoded by uORF1, uORF4 or the nascent protein encoded by *GCN4*.	i = 239 (uORF1 polypeptide released);<br>i = 449 (uORF4 polypeptide released);<br>i = 1434 (Gcn4 released);<br>i = 1534 (3'-end, no product).	32.7 (15)
6	Ribosomal termination with ribosome remaining associated with mRNA	80S(i) → 40S(i) + translation product	Translation product is the polypeptide encoded by uORF1.	i = 239 (uORF1 polypeptide released).	32.7 (15)
7	translation elongation	80S(i) → 80S(i+3)		If the next codon (i+3) is unoccupied.	Obtained from (15).

Taken together, these possible reactions constitute a system that embodies the following process:

1. At the very beginning, a ribosome loads onto the mRNA.

2. Before uORF1 (230 nt), the ribosome scans at a constant rate of 30 nt/sec.

3. When it reaches uORF1, the ribosome recognises AUG triplet at a constant rate.

4. uORF1 is translated, the translation rate of each codon is obtained from (15).

5. After uORF1 translation, the 40S ribosomal subunit may remain bound to the mRNA at 50% probability.

6. The 40S ribosomal subunit scans the rest of mRNA at a constant speed of 30 nt/sec. It may reaquire a TC during scanning.

7. The 40S ribosomal subunit (with or without TC) scans before reaching uORF4 (440 nt) at a constant speed of 30 nt/sec.

8. If the 40S ribosomal subunit acquires TC before reaching uORF4 (440 nt), uORF4 is translated. After uORF4 translation, the 40S ribosomal subunit dissociates from the mRNA.

9. If the 40S ribosomal subunit acquires TC after it reaches or passes the start codon of uORF4 but before GCN4, GCN4 is translated.

10. If the 40S ribosomal subunit (with or without TC) eventually reaches the 3'-end of mRNA (1534 nt), it aborts scanning.

For list of references, please see the main text.
